# Supplementary material for: Are carbon emissions trading and green financial instruments synergistic? -Comprehensive quantitative research based on content analysis
Source: PLoS One. 2024 Mar 7;19(3):e0298601. doi: 10.1371/journal.pone.0298601 (PMC10919601; doi:10.1371/journal.pone.0298601)
Supplement: S1 Data — (ZIP) [file pone.0298601.s001.zip › Data/地级市Z_F工作报告.zip!/╦─┤¿/╣π╘¬╩╨/└┤╫╘╣1⁄2╓┌║┼í╛┬φ┐╦╩2╛▌═°í┐_2018.docx]

各位代表：

现在，我代表市人民政府向大会报告工作，请予审查，并请各位政协委员提出意见。

2017年工作回顾

2017年，在省委、省政府和市委的坚强领导下，在市人大、市政协的监督支持下，全市上下齐心协力、砥砺奋进，全面实施“三个一、三个三”兴广战略，预计可圆满完成市七届人大二次会议确定的目标任务。

——经济运行稳中向好。地区生产总值预计实现730亿元，增长8%。规模以上工业增加值预计增长9%、主营业务收入增长19%、利润总额增长51%。居民消费价格预计上涨1.6%。城乡居民人均可支配收入预计分别增长9.1%、9.9%。地方一般公共预算收入完成44亿元，同比增长13%。网供电量首次突破50亿千瓦时，1-11月工业用电量增长10.1%，居全省第5位。

——脱贫攻坚再战再捷。利州区通过摘帽验收，255个贫困村退出，6.78万贫困人口脱贫，贫困发生率下降到4.1%。多项工作经验在全国全省交流，全国易地扶贫搬迁现场会、产业扶贫现场会，全省贫困县摘帽现场会、社会扶贫推进会等在我市召开。

——项目投资高速增长。全社会固定资产投资扭转多年低速增长局面，预计完成720亿元，增长16.4%，比上年提高10.4个百分点，是八年来最高增速。200个重点项目完成投资占固定资产投资的51.4%。总投资270亿元的中青城投昭化古城旅游度假区和142亿元的广平高速公路开工建设，是建市以来投资额最大的单体项目和交通项目。

——产业结构加快调整。预计一二三产业增加值分别实现114.6亿元、335.8亿元、279.6亿元，比重由16.1︰46.6︰37.3调整为15.7︰45.9︰38.4。战略性新兴产业产值增长20%，高新技术产业产值实现190亿元。

——动力活力持续释放。农村“两权”抵押贷款、山区农业供给侧结构性改革等改革经验在全省推广。市文旅集团挂牌成立。建成公用型保税仓库，中欧班列组货基地加快建设。成广合作扎实推进，签约项目140个、落地63个。大众创业、万众创新蓬勃发展，预计新增各类市场主体2.65万户，增长18.6%。预计科技进步对经济增长的贡献率达48.2%。

——服务效能显著提升。大力开展“马上办”提速行动，深入推进“三减一转两提高”，明确“最多跑一次”清单事项152项，政府系统公文网上办理达90%以上。简政便民专项行动取得实效，市民“10分钟缴费圈”基本形成。

兰渝铁路、西成高铁全线开通运营，打开了人才、资金、信息、技术流动的“高速通道”，广元发展进入“高铁时代”。

一年来，主要做了以下工作:

一是下足“绣花”功夫，全力决胜脱贫攻坚。以脱贫攻坚统揽经济社会发展全局，健全作战指挥体系，出台精细化管理30条规程，建立返贫预警监测阻击机制，加大“插花”贫困户帮扶力度，开展全覆盖巡回督查暗访，逗硬监督执纪问责“三项制度”，加强民主监督、督促检查、考核奖惩。开展“四讲一做”、“同吃同住同劳动”等活动，群众主体作用、感恩意识和满意度增强。6万贫困群众住上好房子，5.36万贫困学生得到资助，34.82万贫困人口享受医疗救助，5.85万贫困人口享受兜底扶持，3.9万贫困人口安全饮水问题得到解决。在全省率先全面开展“政担银企户”财金互动扶贫试点。金融精准扶贫贷款余额204.83亿元，增长109.1%。广泛汇聚社会力量，社会扶贫投入16.5亿元。

二是多措并举综合施策，全力稳定经济增长。抓“项目年”稳增长。创新项目策划包装和推进机制，滨江西路、凉水泉风电场等17个重点项目竣工，西二环一期、万贯五金机电城等77个重点项目加快建设，北二环东延线、有色金属循环与综合利用等83个重点项目开工建设，嘉陵江摆宴坝大桥、川陕苏区红军文化园等27个重点项目前期工作顺利推进，127个项目纳入全省川陕革命老区振兴发展重点项目库。集中开工项目6批次，总投资1106.5亿元，增长103.8%。主动对接国家、省重点投向，争取到位各类资金204.4亿元，增长11.2%。推进政府和社会资本合作，新增入库项目30个，落地项目21个。抓消费稳增长。开展市场拓展“三大活动”145场次，推动“广元造”产品销售近8亿元，广元产品本地市场占有率进一步提高。扩大康养、电商等新型消费和服务供给。社会消费品零售总额预计实现372亿元，增长12.5%。抓服务稳增长。坚持工业企业、外贸出口企业、重点投资项目、民营企业困难问题会商会制度，启明星铝业实现满负荷生产并扭亏为盈，旺苍攀成钢焦化与铁路达成量价互保，煤焦化产业链实现达产达效。制定涉企优惠政策清单，用好工业企业应急转贷资金，出台财金互动支持实体经济和促进民间投资等政策，预计民间投资增长15.5%。

三是大力促进产业转型，持续增强发展后劲。把产业发展作为跨越发展的最大支撑，逐行业逐领域专题研究，出台政策文件近20个。始终坚持工业强市。坚持做大总量与转型升级并重，娃哈哈第二生产基地、中哲新材料二期等232个项目开工建设，剑兴锂电2亿安时锂离子电池技改扩能、日航机电产业园等项目加快推进，新纪元电动汽车动力总成一期、蜀塔稀土铝合金电缆等227个项目建成投运，工业投资、技改投资预计分别增长12%、10%。聚力五大优势产业，提升战略性新兴产业，产值预计分别占工业总产值的66%、16%。加快建设清洁能源利用基地，省燃气公司在广元成立。培育全省大企业大集团3户，预计新增规模以上工业企业40户。推进七盘关国际石材城、普安工业园等园区建设，新增园区面积4500亩。广元经开区获批国家园区循环化改造示范试点园区，利州区成为全省工业强县示范县。建筑业增加值预计增长10%，新增一级资质建筑企业1家。

加快发展现代服务业。图腾·海博春天等商贸项目竣工投用，金橄榄广场、川北国际汽车城等商贸物流项目加快建设。积极培育地方特色餐饮。推动电子商务与一二三产业融合发展，国家电子商务进农村示范县扩大到4个。大力推进中国生态康养旅游名市建设，华侨城剑门关旅游区、康养示范产业园等一批重大项目落地，成功举办低碳发展与生态康养旅游名市建设国际论坛、省第二届生态旅游博览会，荣获全国森林旅游示范市称号。新增1个国家农业公园、3个国家4A级旅游景区。全年接待游客4400万人次、旅游总收入330亿元，分别增长16%、25%。加快完善金融体系，创立广元农商银行、剑阁农商银行，引进长城华西银行、雅安商业银行。预计银行业金融机构存贷款余额分别增长9.5%、12.5%，存贷比提高1.8个百分点，保费收入增长10.5%。金融生态环境评价居全省第7位。新增新三板挂牌企业2家，四板挂牌企业13家。

深入推进农业供给侧结构性改革。坚持“三园联动”建基地，新建现代农业园区7个、村特色产业示范园782个、户办产业小庭园6.99万个，新增特色产业基地16万亩，培育各类新型农业经营主体7648家。肉牛羊、剑门关土鸡和生态渔业发展取得新进展。粮食产量实现“十一连增”。推动产业延链搞加工，累计建成农产品产地初加工中心50个，发展规模农产品加工企业169户。突出绿色有机创品牌，“三品一标”农产品达338个。被确定为国家农产品质量安全示范市创建试点，启动创建全国有机产品认证示范市。“广元七绝”成为全省十大优秀农产品区域公用品牌。特色农产品出口增长228.4%。曾家山成为国家农旅文融合示范区，苍溪县成为中国特色农产品优势区和国家畜牧业绿色发展示范县。

四是深化创新改革开放，不断凝聚发展动能。审议出台58个专项改革方案，深入推进9大重点领域改革。纵深推进“放管服”改革，完成行政权力责任清单动态调整，市本级行权事项减少796项。事业单位分类改革有序开展。一体化政务服务平台基本建成。在全省率先开展“双随机一公开”联合检查。全面实施“多证合一、一照一码”登记制度改革，规范涉企收费和涉审中介管理。开展“双提双破”专项行动，发展环境进一步优化。推进农村集体产权制度改革，累计完成1579个村清产核资和集体经济组织成员认定。推进农村土地“三权分置”，引导土地经营权规范流转。启动市属经营性国有资产集中监管，实行市属国有企业分类监管。推进去产能去库存降成本，关闭煤矿11家、退出产能99万吨，房地产去化周期控制在10个月以内；扩大直购电、富余电量消纳政策惠及范围，减少企业电费支出4亿余元；落实减税降费，减轻企业负担18亿元以上。广北二专线管理权下放，停止设站收费。被确定为全省促进科技与金融结合综合试点市。新增国家高新技术企业5家、省级院士（专家）工作站2家，实施科技成果转化项目158项，申请专利1600件。推进军民融合，军民融合产业产值预计实现77亿元，增长6%。深入推进大开放大合作，积极融入“一带一路”。加大成都、深圳等地产业转移承接力度。改革投资促进体制机制，全面修订招商引资优惠政策，增强招商工作竞争激励和市场化水平。积极参加和举办各类投资促进活动，预计引进到位市外资金548亿元，增长19.7%。加强返乡创业服务，新增返乡创业3786人，创办经济实体3605个，带动新增就业1.36万人。

五是提高规划建设水平，加快城乡协调发展。推进“多规合一”，市、县、乡三级规划管理体系逐步完善，完成城市总规大纲和控规修编论证评审。推进三江新区、东部新城、北部新城建设，全市新增建成区面积5.5平方公里，城镇化率提高1.5个百分点。完成海绵城市和综合管廊规划，081产业新城等项目加快推进，高水平规划三江新区核心区（宝轮）、黑石坡森林公园。完成盘龙机场停机坪升级改造和南山净空治理，新开通广元—海口航线。广元铁路枢纽总图规划完成，西部第一个地级市动车运用所落户广元。城市交通体系不断完善，绕城高速大石连接线基本建成，中心城区智慧停车场、南山隧道加快推进。拓展“百镇建设行动”，昭化镇入选国家特色小镇，曾家镇入选全国运动休闲特色小镇。城乡建设用地增减挂钩节余指标流转1.1万亩。城市管理执法体制改革稳步推进，“四定”网格管理机制巩固提升。新建幸福美丽新村260个、新村聚居点300个、“1+6”村级公共服务中心260个。创建省级“四好村”112个。夯实农村基础设施，新建通村公路1500公里、渡改桥12座，新建和整治渠道528公里、塘堰528口，新增有效灌面3.1万亩。曲河、双峡湖等中型水库加快建设。

六是贯彻绿色发展理念，加快生态文明建设。认真接受中央、省环保督察，省环保督察反馈问题已整改完成96%，信访件全部办结。深入开展大规模绿化全川广元行动，森林覆盖率达到56.18%。推进中国最干净城市建设，开展扬尘整治、油烟整治、秸秆禁烧、工业治污、机动车污染防治五大行动，主城区环境空气质量优良天数达95.8%。全面落实河长制，主要河流水质达到Ⅱ类以上标准，白龙江达到Ⅰ类标准。在全省第一个完成市城区黑臭水体整治。启动土壤污染详查与修复工作。加强农业面源污染防治，依法关闭或搬迁禁养区养殖场（户），推进废弃农膜收集处理，秸秆综合利用率达到91.8%。强力推进城乡环境综合治理，国家卫生城市复审高分通过。建立环境保护与产业发展协同机制，落实主体功能区制度，在全省率先实行县域间水环境生态补偿。推进国家生态文明先行示范区建设，纳入国家气候适应型试点城市，被评为2017中国绿色发展优秀城市。预计单位GDP能耗下降3.5%，单位工业增加值能耗下降4%。主要污染物年度削减目标全面完成。

七是坚持以人民为中心，全面保障和改善民生。完成十项民生工程和30件民生大事，一般公共预算民生支出占比65%。城镇新增就业3.97万人，城镇登记失业率3.85%。实现劳务总收入188.7亿元。推进全民参保，城乡居民基本养老保险覆盖113.83万人，基本医疗保险参保率达97.5%。筹资22.57亿元全面解决近十年来3.4万被征地农民社保遗留问题。新开工改造城镇棚户区3781套，货币化安置2148户，改造农村危房1.74万户。新改扩建养老机构床位3000张，新增城乡社区日间照料中心69个。建成市城区应急水源工程，白龙水厂加快建设。实施饮水安全巩固提升工程，覆盖农村人口10万人。旺苍县、苍溪县、昭化区通过义务教育发展基本均衡县国家认定，朝天区、剑阁县通过省级评估。加强农村小规模学校建设管理。引进高级教育管理人才11名、特优骨干名师304名。提高高中阶段教育质量，高考本科上线人数创历史新高，回流广元籍高中学生1173人，吸引市外高中生源1020人。市特殊教育学校开工建设。川北幼专通过教育部人才培养评估。推动普职融通试点，组织580名往届初高中毕业生再读职业学校。公立医院改革、医养结合、中医药发展走在全省前列。分级诊疗制度全域实施，家庭医生签约服务全面推进，重大疾病、地方病有效控制。在全省率先出台鼓励二孩生育配套政策，实行孕产妇住院分娩基本医疗免费。实施文化惠民工程，建成255个村文化室，公共文化场馆免费开放率100%。省运会、残运会和特奥会筹备工作顺利开展。推进全民健身，柔力球竞赛套路获第十三届全国运动会铜奖，举办各类群众体育赛事260余次。成功举办省首届体育产业战略发展高峰论坛。成功创建省级文明城市。审计、统计、公积金、人防、防震、气象、老龄、残疾人、红十字会、双拥、慈善、民族宗教、地方志、对台、外事侨务、档案等工作有效开展。

八是创新政府建设和社会治理，确保社会和谐稳定。认真执行市人大及其常委会决议、决定，主动接受人大工作监督、法律监督和政协民主监督，完成代表委员建议提案办理。首部实体性地方法规《白龙湖亭子湖保护条例（草案）》提交市人大常委会审议。完善公职律师制度，实现政府法律顾问全覆盖。支持人民团体有效开展工作。加大“四风”问题监督检查力度，严肃查处违纪违法案件。严查招投标领域违法犯罪案件，整治招投标乱象。健全应急处突机制，妥善高效处置嘉陵江铊污染事件，受到国务院、环保部、省政府充分肯定。安全生产形势总体平稳，事故起数、死亡人数明显下降。加强食品药品监管，开展城区活禽宰杀、餐厨垃圾、医疗器械使用等专项整治，加强农村宴席食品安全监管。积极防范处置非法集资和投融资领域突出问题，有效防控金融风险。深化“法律七进”，深入排查化解矛盾纠纷，推进社会治安防控体系转型升级。严厉打击各类违法犯罪行为，开展“40天平安行动”，公众社会安全满意度第三方测评居全省第1位。

各位代表，过去一年取得的成绩，是省委、省政府坚强领导、深切关怀的结果，是市委加强领导、科学决策的结果，是市人大、市政协有效监督、大力支持的结果，是全市广大干部群众辛勤工作、共同努力的结果。在此，我代表市人民政府，向全市人民，向人大代表、政协委员，向各民主党派、工商联、无党派人士、各人民团体和社会各界人士，向驻广解放军、武警官兵、政法干警和民兵预备役人员，向所有关心支持广元发展的朋友们，表示衷心的感谢!

我们也清醒看到，发展不平衡不充分的矛盾在我市表现十分明显，尤其是发展不充分的问题突出。主要表现在：经济总量小，发展速度和效益仍需提高，工业主导地位不强，人均固定资产投资额在全省排位靠后，财政增收难度较大；脱贫攻坚任务依然艰巨，农民持续增收压力较大，教育、医疗、就业等还不能充分满足人民对美好生活的需要；政务服务水平仍需提高，发展环境仍需改善。

2018年主要目标和重点工作

2018年，是贯彻党的十九大精神的开局之年，是决胜全面建成小康社会、实施“十三五”规划承上启下的关键一年。做好今年政府工作的总体要求是：以习近平新时代中国特色社会主义思想为指导，深入学习贯彻党的十九大和中央经济工作会议、省第十一次党代会、省委十一届二次全会和省委经济工作暨全省金融工作会议精神，全面落实市第七次党代会、市委七届二次、五次、六次全会和市委经济工作暨全市金融工作会议精神，专注发展战略定力，坚持新发展理念，按照高质量发展要求，坚持稳中求进、跨越发展工作总基调，坚定实施“三个一、三个三”兴广战略，以供给侧结构性改革为主线，统筹推进稳增长、促改革、调结构、惠民生、防风险各项工作，打好防范化解重大风险、精准脱贫、污染防治三大攻坚战，为决战决胜整体连片贫困到同步全面小康跨越，加快建设川陕甘结合部现代化中心城市打下坚实基础。

主要预期目标建议：地区生产总值增长8%以上，规模以上工业增加值增长9.5%以上，全社会固定资产投资增长15%以上，社会消费品零售总额增长12%以上，地方一般公共预算收入增长8%以上，城乡居民人均可支配收入分别增长9%以上、9.5%以上，居民消费价格涨幅控制在3%以内，城镇登记失业率控制在4%以内，城镇化率提高1.5个百分点，完成节能减排目标任务。

上述目标的提出，综合分析了我市的发展条件，充分论证了支撑要素，全面考虑了与全国全省同步全面小康的现实需要，符合稳中求进、跨越发展的工作总基调。实现上述目标，必须始终坚持扩大投资，把项目投资作为经济发展最大引擎；始终坚持推进改革创新开放，增强内生发展动力；始终坚持生态优先绿色发展，实现环境保护和产业发展协同推进；始终坚持以人民为中心，在推进改革发展稳定中不断增强群众获得感、幸福感。

第一，着力脱贫攻坚再战再胜。聚焦“两不愁、三保障”和“四个好”，坚持精准方略，实施脱贫奔康“六化”行动，实现青川县、昭化区、朝天区“摘帽”和232个村退出、6.23万人脱贫。

全方位强化精准滴灌。科学制定县摘帽、村退出、户脱贫和行业扶贫计划，整合落实各类项目资金，全面满足脱贫攻坚需求。继续抓好产业就业扶贫、集体经济发展和新型经营主体培育，增强脱贫造血能力，实现多渠道增收。认真落实易地扶贫搬迁、危旧房改造、教育、医疗、社保等扶贫政策，推进交通、水利、电力等基础设施建设，改善群众生产生活条件。加强东西部扶贫协作，深化浙江对广扶贫协作，引导民营企业和社会组织参与扶贫，增强攻坚合力。

全覆盖抓好结对帮扶。坚持“六个一”帮扶机制，均衡配置帮扶力量，同步提升贫困村和非贫困村、已脱贫村帮扶效果。常态化开展帮扶干部与贫困户“同吃同住同劳动”活动，帮助解决实际困难，增强群众对脱贫攻坚的认同感满意度。坚持扶贫同扶志、扶智相结合，通过树立脱贫典型精神激励、建立道德积分物质激励、严格村规民约制度激励，更好发挥群众主体作用。

全过程保持攻坚态势。突出关键指标、重点领域、薄弱环节、边远死角，全面加强督查暗访。对标国家、省考核评估重点，健全考核机制，提高考核评估的科学性实效性。强化考核结果运用，开展扶贫领域作风问题专项整治，严格问责问效，鲜明重实干、重实绩导向。建立完善长效机制，巩固脱贫成效。认真接受民主党派扶贫监督。

第二，全力以赴抓好项目投资。围绕项目工作，组建、充实前期工作、政府和社会资本合作、财政评审、联合审批、图审、项目推进六大中心，千方百计推动项目投资。

高水平谋划争取项目。围绕“一枢纽一名市三基地”目标，在基础设施、产业发展、城乡建设、社会事业等领域谋划储备一批项目，策划包装一批PPP项目，常态化项目储备1万亿元以上。精准对接“一带一路”、革命老区振兴发展、乡村振兴等重大战略和政策机遇，争取更多项目纳入中央、省规划盘子。力争京昆高速广元段扩容改造等重大项目前期工作取得明显进展。

提升项目推进实效。发挥六大中心作用，强化六大机制，认真解决项目具体困难和问题，形成开工一批、推进一批、竣工一批、加快前期一批的有序接替。抓好220个重点项目，确保完成投资400亿元以上。按季度举行集中开工活动，确保动车运用所、七盘关至曾家山旅游扶贫公路等重大项目开工建设。加快建设广平高速、日航机电产业园等重大项目。推动西二环道路、白龙水厂等重大项目尽早竣工。

扎实抓好要素保障。抓好市场融资，加强向上争取，强化资金保障。落实银行业金融机构支持地方经济发展考核与市级可调控资金存放挂钩办法，推进金融创新改革，提高存贷比2个百分点以上。积极引导民营资本参与项目建设。积极争取新增建设用地计划，加快征地拆迁进度和建设用地审批，用好差别化用地政策，优化土地要素保障。加强水、电、气、运等要素协调。持续开展招投标集中治理，继续整治发展环境。

第三，推动产业发展提速提质。全面推动三次产业转型发展、跨越发展，加快构建速度更快、质量更高、效益更好、结构更优的现代产业新格局。

发展壮大新型工业。坚持和强化工业强市，不断提升工业整体实力和核心竞争力。突出发展先进制造业，落实《中国制造2025广元行动方案》，狠抓生物医药、新材料、新能源汽车、新一代信息技术等新兴产业发展，力争高技术产业产值占规模以上工业12%以上。推动食品饮料、机械电子等传统产业调整结构、提档升级。推动军民深度融合，加快建设国家新型工业化产业示范基地。加快工业项目建设，实现工业投资、技改投资较快增长。狠抓骨干企业培育，实施企业上市5年行动计划和中小企业梯度培育计划，力争新增亿元企业10户，培育新进规企业45户。坚持“一县一主业”、“一园一特色”，推进中国西部（广元）绿色家居产业城等重点园区建设，新增园区面积4000亩以上，进一步拓展工业发展空间。

推动建筑业转型升级。培育升级一批建筑企业，引导广元籍域外企业回归，组建广元建工集团。推进建筑节能示范工程建设，新建住宅和公用建筑严格执行节能50%标准。推广应用装配式建筑。力争建筑业产值达到195亿元。

加快发展现代服务业。推进农产品物流中心、川北国际汽车城等重点商贸物流项目建设。推动铁路枢纽综合货场物流基地和高铁枢纽综合配套现代服务园区规划建设。进一步完善市主城区、三江新区和重点集镇商贸体系。大力发展购物中心、超市、便利店和社区商业,完善多层次流通网络。抓好“放心舒心消费城市”创建。优化提升市场拓展“三大活动”，加强“广元造”产品推广应用。多元化发展商业银行、保险、信托、证券、融资担保、融资租赁等金融业态，提升金融服务规模和质量。引导房地产企业向康养、工业、旅游地产转型发展。实施电子商务“四大工程”。

大力发展生态康养旅游业。创建国家全域旅游示范区、国家级医养结合试点市，推进中国生态康养旅游名市建设，打造大蜀道国际旅游目的地。全力推进中青城投昭化古城、华侨城剑门关等重点项目。强化旅游公共服务体系和配套要素建设，推进旅游厕所革命、智慧旅游，开发广元特色餐饮和旅游商品。实施“旅游+”，推进文旅、农旅、体旅融合发展。强化旅游品牌整合营销，开展央视《魅力中国城》等系列营销活动。办好第三十届女儿节。力争接待游客突破5000万人次，实现旅游总收入400亿元。

着力发展现代农业。推进农业供给侧结构性改革，抓好建基地、创品牌、搞加工等重点任务。压实粮食安全主体责任，发展绿色优质粮食产业，切实维护粮食安全。坚持“三园联动”，壮大特色优势产业基地和生态畜禽养殖规模，新建现代农业园区8个。加快发展红心猕猴桃、富硒富锌茶叶、道地中药材等特色优势产业，大力发展肉牛羊、剑门关土鸡和生态渔业。实施以品种改良为主的油橄榄提质增效行动，积极发展笋用竹。加强品牌宣传营销，提升“广元七绝”区域公共品牌影响力。强化龙头企业和新型经营主体培育，建立企业与农户更紧密的利益联结机制。发展农产品加工业和休闲农业，推进一二三产业融合发展。

第四，推进创新改革和开放合作。加快推进创新驱动发展，深入实施大开放大合作战略，让经济发展更具活力和韧性。

扎实推进供给侧结构性改革。推动全国有机产品认证示范市、国家农产品质量安全示范市创建，打造优质高端农产品供应基地。优化信贷投向，加强对实体经济、工业企业、小微企业支持力度。支持企业利用资产证券化盘活存量资产，利用股权和各类债务工具融资。落实减税降费政策，积极争取直购电、富余电量消纳等政策，协调降低铁路运价，进一步降低企业成本。加强质量强市建设，提升“广元造”影响力和市场占有率。

深化重点领域和关键环节改革。完善国有资产管理体制，加强国有企业监管。落实非公有制经济发展政策措施，支持民营经济发展。深化投融资体制改革，完善政府和社会资本合作机制。推进商事制度改革。深化供销社综合改革。深化财税体制改革，厘清基本服务领域财政事权与支出责任。深化农村土地“三权分置”改革，鼓励适度规模经营。推进农村集体产权制度改革，发展壮大集体经济。组建区域性食品药品检验检测中心，为产业发展提供支撑。协同创建川陕革命老区综合改革试验区。

激发创新创业活力。深化市校合作、厅市会商，新签订一批产学研合作协议，大力实施产业技术攻关。推动省级促进科技与金融结合综合试点市建设。加强工程技术研究中心、重点实验室、院士（专家）工作站、海智工作站等创新平台建设。支持发明专利申请，推进技术转移服务体系和中介机构建设，促进科技成果转化。实现高新技术产业产值200亿元，科技进步对经济增长的贡献率达到50%以上。突出“军转民”、“民参军”，加快建设军民融合产业园区和平台。创新人才引进、培育、使用、激励机制，强化人才支撑。

提升开放合作水平。落实投资促进体制机制改革部署，推进招商工作专业化、市场化、社会化。深化成广合作，力争签约落地130个工业项目。扩大与长三角、珠三角、环渤海、成渝经济区交流，深化浙广、九广合作。扩大北向开放，积极参与西北、华北市场分工。深度参与中外知名企业四川行、西博会、科博会等各类招商活动，引进到位市外资金650亿元，增长18.6%。积极融入“一带一路”，加快中欧班列广元组货基地、公用型保税仓库建设，加强与国外、境外交流合作，发展加工贸易，扩大外贸进出口。

第五，全面提升城乡建设水平。建立更加有效的区域协调发展机制，增强区域发展的协同性、联动性和整体性。

加强基础设施建设。推进绵万高速苍巴段、省道411线苍溪至旺苍段一级公路开工建设，加快国道542线广元至巴中段前期工作。完成国省干线大中修工程，推进“四好农村路”建设，建制村通客车率达到90%。加快广元港张家坝作业区、上石盘电航枢纽船闸工程建设，建成嘉陵江航运配套二期工程。开工改造广元机场跑道。建成乐园、曲河、双峡湖3座中型水库，加快建设大寨、雷家河水库和武引二期灌区，加快亭子口大型灌区、罐子坝水库等前期工作。继续实施农村饮水安全巩固提升工程，覆盖农村人口10万人。新建加固堤防10公里，治理中小河流15公里。继续实施“宽带乡村”工程。

提升城镇规划建设管理水平。推进“多规合一”，完成城市总规和控规修编，做好特色小镇规划编制，不断完善城镇体系。加快主城区与三江新区核心区（宝轮）、东部新城、北部新城一体化发展。启动南河片区海绵城市示范项目，加强农贸市场、城市公厕等基础设施建设，加快推进黑石坡森林公园改造提升和川陕苏区红军文化园建设，不断完善城市功能。启动一环路提升改造、中央森林康养大道等项目，加快建设南山隧道、北二环东延线等交通干线和中心城区智慧停车场。抓好国家级、省级重点镇建设，创建一批重点特色小镇。推进城市管理执法体制改革，提升城市管理水平。

强力实施乡村振兴战略。实施全域乡村规划提升工程，启动乡村振兴规划。推进农村人居环境整治，加快土坯房改造，推动建设集循环农业、创意农业、农事体验于一体的田园综合体。抓好第三轮省级新农村示范县和市级示范片建设，建成幸福美丽新村250个、配套完善“1+6”村级公共服务活动中心232个，创建省级“四好村”120个。完善村规民约，树立文明乡风，加强农村社会治理。

做大做强县域经济。夯实县域规划、管理、产业支撑，实现县区差异化、补位化发展，加快建设工业强县、农业强县、旅游康养强县和生态强县，提升县域经济实力。推进宜居宜业县城建设，坚持“产城一体、园城一体”，加快产城融合发展，提高人口吸纳承载和经济辐射带动能力。统筹规划建设城乡公共基础设施，促进城乡公共资源均衡配置。

第六，坚持生态优先绿色发展。树立和践行绿水青山就是金山银山的理念，彰显生态环境优势，积极发展生态经济。

解决突出环境问题。继续抓好环保督察问题整改。着眼建设中国最干净城市，全面推进城乡环境综合治理，坚决打赢污染防治攻坚战。持续整治工业排放、城市扬尘等大气污染，巩固无霾城市建设成果。全面落实河（湖）长制，加强“两湖”流域综合治理。治理农业面源污染，开展土壤环境质量状况详查，加强农用地污染治理。

巩固生态安全屏障。全方位推进绿化、彩化、美化、香化、亮化，完成营造林30万亩。推进国家园林城市创建和蜀道植物动物园建设。推动主体功能区转型发展，积极参与大熊猫国家公园建设。划定并严守生态保护红线，推动建立跨界流域环境保护联动机制。加强林业有害生物防治、生物多样性保护和森林防火。大力实施水土保持和生态修复工程，做好地质灾害防治工作。

推进绿色发展。推动环境保护与产业发展相协同，推广新能源公共交通，开展碳排放权交易，推进国家新能源示范城市、低碳城市建设和城市生活垃圾分类试点。推进秸秆综合利用、养殖粪污治理、病死畜禽集中无害化处理、废弃农膜收集处理、化肥农药减量控害、饲料清洁化等专项行动，促进农业废弃物资源化利用。加强环保基础设施建设和运营维护。倡导绿色生活、绿色消费、绿色出行。

第七，加强民生改善和社会建设。坚持民生投入只增不减，为民办实事只增不减，一件接着一件办，一年接着一年干，不断满足人民日益增长的美好生活需要。

扎实办好民生实事。一般公共预算民生支出占比保持在65%以上。继续实施十项民生工程和30件民生大事。推进高校毕业生、城镇失业人员、就业困难人员等重点群体就业，鼓励创业带动就业，支持返乡创业，确保城镇新增就业3.2万人。推进全民参保计划，抓好社会保险扩面征缴、建筑业按项目参加工伤保险，推动社会保障法定人群全覆盖。深化医保支付方式改革，扩大异地就医即时结算范围。实施百万安居工程建设行动，推进棚户区改造和货币化安置，新开工改造各类棚户区4000套。推进移民库区振兴发展，实现库区长治久安。

全面提升教育质量。实施第三期学前教育行动计划，普惠性学前教育覆盖率达到65%。实施中小学标准化建设，开展教育信息化二期建设，各县区全面通过义务教育均衡发展国家评估。实施消除大班额三年攻坚计划，市城区新建、改建初中2所、小学2所、公办幼儿园4所。着力提高高中阶段教学质量，吸引本地生源回流。多元发展职业教育和技能培训。探索公共财政扶持民办教育发展机制。实施“四名工程”，推进教育领域综合改革。

推进健康广元建设。深化医药卫生体制综合改革，建立完善分级诊疗和现代医院管理制度，完善医疗保障制度。健全市、县、乡、村四级卫生计生服务体系，加强基层医联体建设，推进医院差异化发展。积极引进域外优质医疗资源，加快建设川陕甘结合部区域医疗中心。稳步推进医疗服务价格调整、公立医院人事薪酬制度改革。加强公共卫生服务和重大传染病、职业病防控，实施全民预防保健行动。完善中医药服务体系，建设中医药强市。

繁荣发展文化体育事业。推进现代公共文化服务体系建设，促进均等化标准化。开展“百千万”品牌群众文化活动，创作一批具有本土特色的文学艺术作品。加强传统文化保护，抓好传统村落保护、历史和地方特色建筑保护、历史名人文化传承创新工程等工作。启动创建全国文明城市，提高公民文明素质和社会文明程度。加快培育文化产业，发展新型文化业态。倡导全民阅读，建设书香广元。抓好比赛场馆建设，办好省运会、残运会和特奥会。大力开展全民健身活动。

做好统计、公积金、老龄、工会、青少年、妇女儿童、慈善、残疾人、红十字会、科普、地方志、档案、外事侨务、对台等工作。

打造共建共治共享的社会治理格局。深入推进依法治市，全面推进“七五”普法，巩固提升“法律七进”，推进公共法律服务体系建设。加强城乡社区治理，完善村（社区）务公开、民主管理制度。支持群团组织参与社会治理。从严落实安全生产责任，夯实安全生产基层基础，狠抓重点行业领域安全监管，持续开展打非治违和重点专项整治，坚决防范遏制重特大事故发生。实施最严格的食品药品安全监管，严防严管严控食品安全风险。持续推进“网格化”服务管理、“雪亮”工程建设，完善信访工作制度，及时排查化解社会矛盾，维护公共安全和社会和谐稳定。加大违法犯罪活动打击力度，严打非法集资、金融诈骗等犯罪行为，防范金融风险。加强国防动员能力和人民防空体系建设，做好双拥共建、国家安全、国防教育、保密、民族宗教等工作。做好水文、气象、防震、防汛抗旱、应急管理等工作。

第八，改进和加强政府自身建设。以建设人民满意政府为目标，不断推进法治政府、高效政府、服务型政府和廉洁政府建设。

坚持依法行政。严格按照法定权限和程序履行职责，坚持依法科学民主决策，严格规范公正文明执法。认真执行人大及其常委会决议、决定，自觉接受人大法律监督和工作监督、政协民主监督，办理好代表委员建议提案。主动接受社会和舆论监督，认真听取人大代表、政协委员、民主党派、工商联、无党派人士和各人民团体意见。自觉接受司法监督。加强审计监督。

提升工作效率。严格执行工作责任制，对重点任务铆紧各方责任、层层传导压力，确保落实到位。持续开展“双提双破”专项行动，进一步完善“马上办”制度机制，加强政务督查，整治“中梗阻”和庸政懒政怠政行为。健全完善正向激励机制和容错纠错机制，给改革创新者撑腰鼓劲。

改进政务服务。推进“放管服”改革，继续清理规范行政权力，建立完善权责清单。全面推行“双随机一公开”联合检查，强化企业信用信息共享交换平台运用。推进“互联网+政务服务”，进一步降低制度性交易成本。提升公用企事业单位服务质效。加快公共资源交易平台建设，强化公共资源交易监管。

加强廉政建设。严格落实“一岗双责”，把全面从严治党、从严治政各项规定落实到政府工作全过程。推进“两学一做”学习教育常态化制度化，开展好“不忘初心、牢记使命”主题教育。深入贯彻落实中央八项规定、省委省政府十项规定和市委“六个带头”精神，坚决防止“四风”反弹。严肃整治侵害群众利益的不正之风和腐败问题，用好阳光问政平台，管好惠民资金和项目，防止寻租腐败。

各位代表！新时代赋予新使命，新征程需要新作为。让我们紧密团结在以习近平同志为核心的党中央周围，以习近平新时代中国特色社会主义思想为指导，在省委、省政府和市委的坚强领导下，大力弘扬“红船精神”和“红军精神”，不忘初心、牢记使命，为决战决胜整体连片贫困到同步全面小康跨越、加快建设川陕甘结合部现代化中心城市而努力奋斗！
